# Complete Genome Sequence of Bacteriophage IndyLu, Isolated from a Microbacterium foliorum Culture

**DOI:** 10.1128/MRA.01079-21

**Published:** 2021-12-16

**Authors:** Ashley Suris, Selina Alvarado, Tommy Butler, Carlos Canales, Matthew Castro, Julia Gaston, Marlee Goppert, Raylon Huckaby, Jesse Laposky, Jessica Lee, Elizabeth Mullins, Damla Ustundag, Josue Zuniga, Faith Cox, Dustin Edwards

**Affiliations:** a Department of Biological Sciences, Tarleton State University, Stephenville, Texas, USA; Queens College CUNY

## Abstract

Microbacteriophage IndyLu was isolated from Microbacterium foliorum NRRL B-24224. The 41,958-bp double-stranded DNA genome has 71 predicted protein coding genes and 1 tRNA. The lytic actinobacteriophage was extracted from soil samples collected in Stephenville, TX, and is related to cluster EB bacteriophages Didgeridoo and Lahqtemish.

## ANNOUNCEMENT

**FIG 1:**
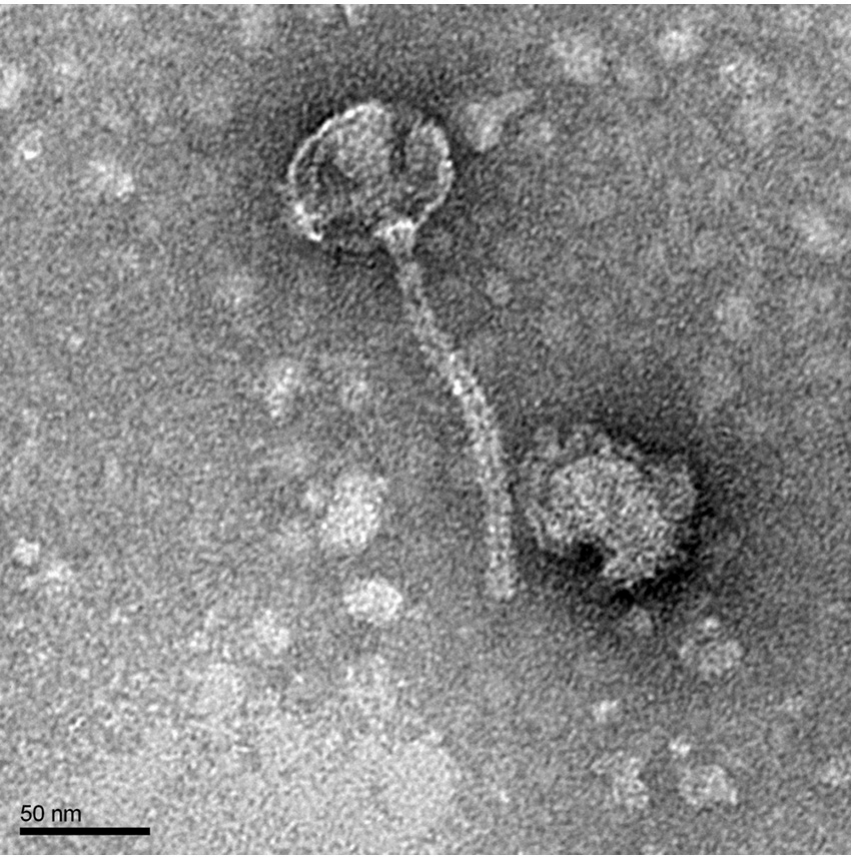
Transmission electron microscopy of cluster EB bacteriophage IndyLu. High-titer lysate was attached to a 300 copper mesh grid and negatively stained with uranyl acetate. Imaging using an FEI Tecnai G2 Spirit BioTWIN transmission electron microscope (NL1.160G) revealed a capsid diameter of 65 nm, tail length of 155 nm, and a morphology consistent with other *Siphoviridae* family members.

Microbacterium foliorum NRRL B-24224 is a rod-shaped Gram-positive aerobic bacterium from the order *Actinomycetales* that contains no intact prophages or apparent antibacteriophage restriction-modification or CRISPR systems (1). Here, we report the whole-genome sequence of actinobacteriophage IndyLu (2), collected from a dry soil sample near a horse barn in Stephenville, TX, USA (global positioning system [GPS] coordinates, 32.248 N, 98.209 W). Soil samples were suspended in peptone-yeast extract-calcium (PYCa) medium and incubated for 2 h in a shaking incubator at 29°C and 200 rpm. The supernatant was centrifuged and filtered through a 0.22-μm filter. Filtrates were plated with the isolation host Microbacterium foliorum NRRL B-24224 using a soft agar overlay. Bacteriophages were isolated by two rounds of picking a single, well-separated plaque, followed by diluting the bacteriophage sample in a 10-fold dilution series and plating with *M. foliorum*. IndyLu formed small, lytic plaques. Negative-staining transmission electron microscopy (Fig. 1) showed a siphoviral morphology. ImageJ v1.53m (3) measured a tail length of 155 nm and a capsid diameter of 65 nm.

High-titer lysates were prepared from flooded plates as described in the *Phage Discovery Guide* and used to extract bacteriophage DNA using a modified zinc chloride precipitation method (4, 5). The Pittsburgh Bacteriophage Institute prepared the sequencing library from genomic DNA with the NEBNext Ultra II kit (New England Biolabs, Ipswich, MA) using an Illumina MiSeq instrument (6), with 1,156-fold coverage and 445,388 total single-end 150-bp reads. A single bacteriophage contig was assembled using the default settings in Newbler v2.9. Quality control checks for assembly, precision, and genome termini were performed using Consed v29.0 (6, 7). The linear viral genome comprises 41,958 bp (G+C content, 66.2%) with a 3′ single-stranded terminal overhang 10 bp long of 5′-ACTCCCGACA-3′.

Whole-genome alignment with NCBI BLASTn (https://blast.ncbi.nlm.nih.gov/) (8) showed greater than 93% nucleotide sequence identity to cluster EB bacteriophages Didgeridoo (GenBank accession number MH045566) and Lahqtemish (GenBank accession number MT889392). Auto-annotation using GLIMMER v3.02 (9) and GeneMark v2.5p (10, 11) was manually refined using Phamerator (12), DNA Master v5.23.2 (http://phagesdb.org/DNAMaster/), and PECAAN. Microbacteriophage IndyLu is predicted to contain 70 protein-coding genes and 1 tRNA coding for glutamine, identified using ARAGORN v1.2.38 (13) and tRNAscan-SE v2.0 (14). Putative functions were assigned to 32 of the 71 protein-coding genes using HHpred (15, 16) and NCBI BLASTp (8). All tools were run with default parameters. Sixty-seven genes are encoded rightwards, including virion structural and assembly proteins, DNA primase/polymerase, endolysin, Cas4 family exonuclease, and two HNH endonucleases. IndyLu is among the 6.8% of cluster EB bacteriophages coding for a tRNA.

### Data availability.

The actinobacteriophage IndyLu genome sequence is available in GenBank under accession number OK318958. The raw reads are available in the SRA under accession number SRX12683423.
